# A Biobanking System for Diagnostic Images: Architecture Development, COVID-19–Related Use Cases, and Performance Evaluation

**DOI:** 10.2196/42505

**Published:** 2023-12-21

**Authors:** Giuseppina Esposito, Ciro Allarà, Marco Randon, Marco Aiello, Marco Salvatore, Giuseppe Aceto, Antonio Pescapè

**Affiliations:** 1 Bio Check Up Srl Naples Italy; 2 Department of Advanced Biomedical Sciences University of Naples Federico II Naples Italy; 3 Faculty of Engineering Free University of Bozen-Bolzano Bolzano Italy; 4 IRCCS SYNLAB SDN Naples Italy; 5 Department of Electrical Engineering and Information Technology University of Naples Federico II Naples Italy

**Keywords:** biobank, diagnostics, COVID-19, network performance, eHealth

## Abstract

**Background:**

Systems capable of automating and enhancing the management of research and clinical data represent a significant contribution of information and communication technologies to health care. A recent advancement is the development of imaging biobanks, which are now enabling the collection and storage of diagnostic images, clinical reports, and demographic data to allow researchers identify associations between lifestyle and genetic factors and imaging-derived phenotypes.

**Objective:**

The aim of this study was to design and evaluate the system performance of a network for an operating biobank of diagnostic images, the Bio Check Up Srl (BCU) Imaging Biobank, based on the Extensible Neuroimaging Archive Toolkit open-source platform.

**Methods:**

Three usage cases were designed focusing on evaluation of the memory and computing consumption during imaging collections upload and during interactions between two kinds of users (researchers and radiologists) who inspect chest computed tomography scans of a COVID-19 cohort. The experiments considered three network setups: (1) a local area network, (2) virtual private network, and (3) wide area network. The experimental setup recorded the activity of a human user interacting with the biobank system, which was continuously replayed multiple times. Several metrics were extracted from network traffic traces and server logs captured during the activity replay.

**Results:**

Regarding the diagnostic data transfer, two types of containers were considered: the Web and the Database containers. The Web appeared to be the more memory-hungry container with a higher computational load (average 2.7 GB of RAM) compared to that of the database. With respect to user access, both users demonstrated the same network performance level, although higher resource consumption was registered for two different actions: DOWNLOAD & LOGOUT (100%) for the researcher and OPEN VIEWER (20%-50%) for the radiologist.

**Conclusions:**

This analysis shows that the current setup of BCU Imaging Biobank is well provisioned for satisfying the planned number of concurrent users. More importantly, this study further highlights and quantifies the resource demands of specific user actions, providing a guideline for planning, setting up, and using an image biobanking system.

## Introduction

The development of internet and communication technology (ICT) has shown a continuously accelerating pace over the last few decades; however, the recent COVID-19 pandemic outbreak prompted a sudden spike in ICT adoption due to social distancing and consequent remote working necessities, including wider adoption of telemedicine with different specifications [1]. New proposals in ICT development in medicine range from outpatient monitoring systems [2] to privacy-preserving infection tracing systems [3], along with the automatic management and enhanced use of patient digital data [4]. Zhang et al [5] demonstrated that chest computed tomography (CT) combined with analysis by uAI Intelligent Assistant Analysis System (a deep learning–based software) can accurately evaluate pneumonia in patients with COVID-19. Typical CT features of COVID-19 pneumonia include bilateral multifocal ground-glass opacities, with the most common site of infection being the dorsal segment of the right lower lobe. Owing to the intelligent assistant’s ability to locate and quantify regions of infection from CT scans, clinicians are aided and guided in diagnosing COVID-19 quickly and accurately. Other studies have shown how CT imaging on patients with suspected COVID-19 pneumonia could support the reverse transcription-polymerase chain reaction test when the result is not reliable [6-8]. These and other works have thus demonstrated the importance and reliability of diagnoses made downstream of chest CTs for patients with suspected COVID-19 pneumonia, along with the need to find alternative ways to share and store patient digital data. Digital health data include diagnostic images, health records, and reports. These data can be additionally associated with information derived from other tests (clinical, genomic, oncological) or quantitative data extracted from the direct processing of images.

The first virtuous initiatives to address this issue were proposed by The Radiological Society of North America [9], European Institute for Biomedical Imaging Research [10], and The Cancer Imaging Archive [11]. These organizations share open-access COVID-19 data sets containing clinical data and diagnostic images that are usually stored in Digital Imaging and Communications in Medicine (DICOM) format with the scientific community. The main objective of these organizations is to provide publicly available data sets to aid research on the prevention and treatment of complex diseases. This objective is also shared by imaging biobanks, which are new study-based platforms used for the collection, distribution, and repository of pseudoanonymized biomedical images derived from CT, magnetic resonance imaging, and nuclear imaging [12,13]. The ICT repositories dedicated to digital biobanking allow researchers and clinical practitioners to consult, annotate, segment, process, and manage collections of medical images and clinical data [14-18]. By promoting standardization and interoperability, these imaging biobanks can be connected to traditional health care information systems such as hospital information systems, radiology information systems, and picture archiving and communication systems [19-21]. The application of common standards and the validation and benchmarking of ICT infrastructures used for the repository architecture is a major focus of the research activity in this field [22]. Indeed, availability of different (role-specific) image access methods and perceived performance (speed of upload) have been confirmed [23] to impact the perceived usefulness, and thus the actual adoption, of such systems.

In line with these necessities, we designed and performed a benchmarking procedure for a biobank of diagnostic images based on open-source technologies. The knowledge of network performance is paramount to planning and monitoring (ie, to explain and guarantee user quality of experience) and is instrumental for the choice of deployment infrastructure [22]. Gaining an understanding of the processing and memory footprint of common usage of the system allows for planning for new deployments. Moreover, continuous monitoring of the usage enables preventing, detecting, mitigating, and resolving performance bottlenecks that would impact the operators. Modern multitier containerized architectures are instrumented with monitoring utilities to collect instantaneous resource consumption [24]; however, linking these measurements to user activities (and thus to user-perceived performance) is far from a trivial task [25]. In this study, we performed an experimental evaluation of an implementation of the digital biobank Bio Check Up Srl (BCU) Imaging Biobank (BCU-IB), derived from the Extensible Neuroimaging Archive Toolkit (XNAT) open-source platform [26] operated by BCU. BCU-IB has been part of the Biobanking and Biomolecular Resources Research Infrastructure-Education Information Resources Center (BBMRI-ERIC), and its national node BBMRI.it. Image collections have been included in the BBMRI-ERIC directory, including those from cohorts of patients with COVID-19. The biobank is currently operational with 10 connected users. Over the years, we expect to increase the number of projects carried out and consequently the number of connected users.

For this analysis, we adopted the viewpoint of the user, focusing on the perceived performance (in terms of transfer completion time) for the most frequent and most network resources–demanding activities (ie, upload and download of diagnostic image sets). Moreover, we consider the viewpoint of server resource consumption, in correspondence to the activities of two types of frequent users: researchers and radiologists. In the considered usage cases, users accessed the biobank of images to study a cohort of patients with COVID-19 collected during the pandemic. We model the user of the biobank as a researcher who uses the biobank as a research tool for analyzing the CT images of interest and as a radiologist who uses the biobank as a viewer of the CT slices and as an annotation tool.

Three different network scenarios were considered: (1) local area network (LAN), as an organization-local network; (2) virtual private network (VPN), with remote secure access through the internet; and (3) wide area network (WAN), which is similar to a VPN but without internet protocol–level tunneling. The local scenario is used as a reference for the performance experienced over the (uncontrolled) internet. As the user-perceived performance can depend on the specific network path between the user and the server, we validated the (uncontrolled) WAN and VPN network setups to represent a “well-behaved and well-performing” network (ie, ample capacity, limited latency, and stable routing). In this way, the measured system performance is mainly due to system dimensioning, constituting a more challenging evaluation for the system (if network path conditions are worse than ideal for one or more users, the system resources are available to serve more users). Regarding network resources, the results highlight that the different upload/download activities exhibit significantly different performance figures. Regarding server resources, we identify different specific user activities that are computationally more demanding. Overall, the analyses confirm the usability of the system and the correct dimensioning of the network and server setups, also pointing to further analyses and future developments. A preliminary version of this work has been presented as a conference paper [27], focusing only on the network performance aspects in a simpler experimental setup. To the best of our knowledge, our work is the first to analyze the resource footprint of a production imaging biobank in relation to common usage by human operators, while associating the measured performance with user activities. Moreover, by considering operations and data sets currently used for COVID-19 research, the results reflect a timely and intensively investigated subject.

## Methods

### System Description

**Figure 1:**
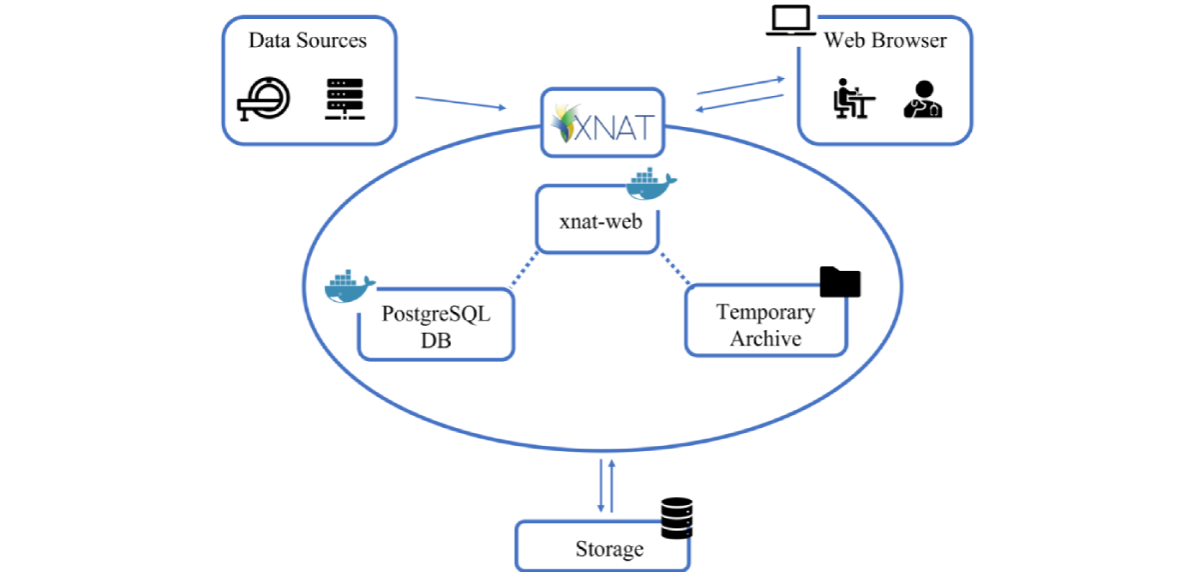
Overall architecture of the system. DB: database; XNAT: Extensible Neuroimaging Archive Toolkit.

There are minimum requirements that a well-structured imaging biobank should satisfy. First, the storage system should be stable and robust, allow for secure data access, and minimize the loss of data. Second, the infrastructure should have a stable internet connection that allows for fast file transport from one system to another. Finally, the system should have all the necessary documentation for processing of the personal data of patients who make their exams available for this purpose. After having fulfilled all these requirements, the installation of the XNAT platform was carried out using lightweight virtualization technologies (Docker containers), according to current best practices to ease the scalability and maintainability of software systems [28]. Specifically, XNAT v1.7.6 was installed using Docker v19.03.14 [29] on the Ubuntu 18.04.5 LTS operating system. The computing and memory resources are as follows: dual central processing unit (CPU) Intel(R) Xeon(R) Silver 4208 CPU 2.10 GHz (8 cores/16 threads per socket for a total of 16/32 cores), 64 GB RAM. Figure 1 shows the main components of the system, including the data sources (eg, picture archiving and communication system, diagnostic devices, external tools, plugins) at the upper left side, the XNAT application containers inside the ellipses, the storage system that provides storage volumes to the XNAT components at the bottom, and the user web interface at the upper right side. The storage is composed of a PostgreSQL Database and a temporary archive on iSCSI volumes provisioned by a storage area network (SAN) connected over 2 10 GB/second ethernet link; the SAN uses 30 10-k rpm disks configured as a RAID 6 array.

### Use Cases

#### Recruitment

The first use case does not involve a user-interface interaction and is associated with the (periodic) loading of diagnostic data; the other two cases are interactive and involve (1) searching and downloading of patients’ diagnostic images and (2) online inspection and annotation of diagnostic images. Each use case is described in further detail below.

In the interactive cases, users access the imaging biobank to study the cohort of COVID-19 patients that has been collected during the SARS-CoV-2 pandemic [30]. Patients from this collection are examined using morphological imaging (thorax X-ray radiography and CT).

#### Diagnostic Data Transfer

A common activity in the life of an imaging biobank is the enrichment of the archived collections of diagnostic data. These uploads can be fully automated or can be manually initiated, but generally do not require high interactivity with the biobank. Several research groups have uploaded data sets of diagnostic images and clinical data on the BCU-IB, contributing to the population of the collections catalogue. The computing and memory footprint of the main components of the XNAT platform have been monitored during these activities to enhance the results reported in previous work and relate them to newly presented tests.

#### Researcher and Radiologist Access

**Figure 2:**
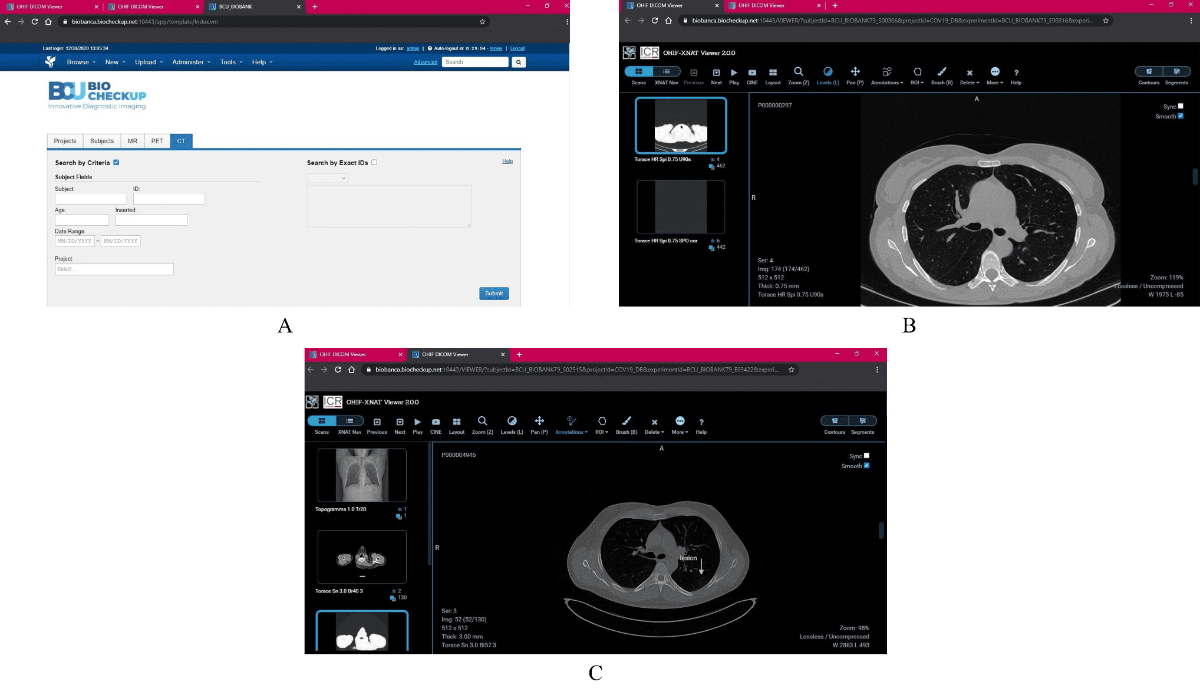
Screenshots of the interactive case studies. (A) Search filter interface for the researcher use case. (B, C) Image viewer during the annotation process in the radiologist use case.

In contrast to the previous case, we investigated the controlled reproduction of interactive access to the biobank. For these tests, we considered the COVID-19 CT collection, which consists of CT scans and clinical data belonging to patients with COVID-19 showing typical patterns of viral pneumonia, such as ground-glass opacity, crazy-paving pattern, and consolidation [31]. This data set is useful for clinical, educational (eg, for training of radiology residents), and technological purposes, such as the implementation of software systems for computer-aided diagnosis [32,33]. In this setting, a researcher user accesses the biobank (LOGIN) to perform a search (FILTER & SEARCH) and the consequent download of a single CT exam (DOWNLOAD & LOGOUT) composed of two series for a total dimension of 459.9 MB (representative of a typical lung CT exam). Figure 2A shows a screenshot of the search filter interface. Once the search result has been obtained, the researcher performs a download of the related data set. The second case of interactive access to the biobank considers a radiologist user who acts as data curator and is in charge of performing the manual annotation of lung lesions induced by COVID-19 on a CT volume set selected from the above-mentioned collection. In this use case, a specific set of images is selected (FILTER & SEARCH) and visually explored (OPEN VIEWER) by scrolling the slices to focus on regions of interest (CHANGE SCAN, PLAY/STOP). No explicit download is performed in this case. Figure 2B and Figure 2C show two screenshots of the image viewer during the annotation process.

### Ethical Considerations

The BCU-IB data collection was carried out under the research protocol approved by the Comitato Etico “Campania Centro,” Italy (protocol number 562/C.E 45/2020 OSS; December 15, 2020) and following the principles of the Declaration of Helsinki. 

### Measurement Metrics

To characterize the performance experienced by the user in the considered scenarios and network setups, along with the load on the system hosting the BCU-IB application, the following metrics have been considered: total transfer duration, average byte rate, CPU percent usage, and memory usage.

Total transfer duration is the main metric, representative of the experience of the user of the digital biobank. Indeed, any transmission issue causing delays and packet losses (in wired connections, as in the considered setups, mostly due to congestion) are surfaced to the user only as an additional delay in completing the transfer. The total transfer duration is computed from the raw packet trace as the difference in time between the latest packet seen and the first one seen. This metric is strongly dependent on the volume of data to be transferred.

The impact of the network conditions on the performance experienced by the user is described through the average byte rate (ie, throughput), calculated as the total amount of transmitted traffic (at the network layer) divided by the duration of the transfer. This metric highlights the efficiency of the network, regardless of the application layer protocol overhead [34]. Therefore, it can be more directly related to the nominal network transfer rate to check if the sending/receiving access connection is a bottleneck for the transfer. Notably, this metric conflates all network-layer-and-up protocols overhead with the user data, including possible retransmissions due to lost packets and time-outs. For this reason, we monitored retransmissions, as they can be inferred from the packet trace captured at the client side.

Measured on a per-container basis, the CPU percentage measures the average utilization over a (small) time interval, representing the percentage of time the container has been using one CPU (not being idle). This value can be *higher than* 100%, as it is normalized *to one CPU processing core*: in the case multiple cores are involved, the average utilization can increase to N×100%, where N is the total number of cores available to the container. If a container saturates CPU usage, then its performance becomes CPU-bound (ie, processing power becomes a bottleneck).

Also measured on a per-container basis, the memory usage measures the instant total occupation of RAM memory. This amount does not include cache memory (which is used to improve performance, but is not strictly required to the execution and can be freed if necessary). The maximum is set for each container (and ultimately is upper-bounded by the physical RAM available on the host machine). For monitoring purposes, memory usage is conveniently expressed as a percentage on the container-allowed memory limit; however, in this work, we report it as the actual byte volume to facilitate the comparison between different containers.

### Experimental Setup

#### Network Setups

**Figure 3:**
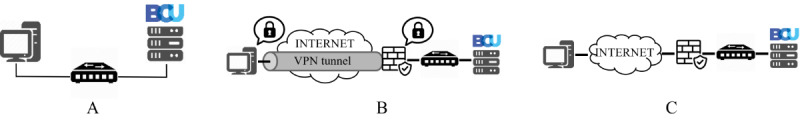
Network setups considered in the experimental evaluations. (A) Local area network (LAN) setup. (B) Virtual private network (VPN) setup. (C) Wide area network (WAN) setup.

Three tests, based on different network setups to represent various deployments or for performance comparison purposes, were designed and carried out as single-user tests, leaving concurrency analysis for future works. All the resources, including internet connections, were not entirely dedicated to this single-user test, but shared with other services and BCU staff works. No other high resource–demanding process or work was conducted during the testing. Each network setup, with the associated significance for a real usage scenario, is described in Figure 3.

Figure 3A represents the LAN setup. We have a client machine that communicates via the LAN (switched Gigabit Ethernet) with the XNAT platform. This scenario was used to investigate the performance on the local network typical of access from inside the laboratory that houses the biobank. The setup also constitutes a benchmarking reference for the (more complex and less controllable) VPN and WAN scenarios. Figure 3B illustrates the VPN setup. This setup is composed of a client machine located on an external network to represent the case of a user who accesses the imaging biobank from any location using the internet connection, adopting a VPN authorized by the host of the platform to communicate in a secure manner. Client-side, the internet access network is a fiber-to-the-home installation, with a nominal network layer capacity of 100 MB/second in both up- and downstream directions. Server-side, the internet access network is a fiber-to-the-premises installation, with a nominal network layer capacity of 300 MB/second upstream and 500 MB/second downstream. We verified that the network capacity was close to the nominal figures for both end points in both directions. The measured path was constant over the whole measurement period. The path consisted of 9 hops, with the first 5 in the client internet service provider (ISP) network and the remaining 4 in the server ISP network (using two different national ISPs). The round-trip time measured from the client showed an average of 18.2 milliseconds (SD 3.166 milliseconds). The VPN client software is OpenVPN [35], an open-source software implementing a secure socket layer (SSL)-authenticated and encrypted sublayer at the application level. The authentication and encryption algorithms are SHA-256 and AES (256-bit), respectively. The VPN server middlebox is a WatchGuard Firebox M270 (guaranteeing 480 MB/second throughput for VPN traffic, according to specs). According to this setup, neither the client-side internet connection nor the server-side middlebox constitute a bandwidth bottleneck for the communications. Figure 3C shows the WAN setup that is composed of a client machine located on an external network to represent the case of a user who accesses the imaging biobank from any location using the internet connection to communicate to the platform by a secure Hypertext Transfer Protocol over Secure Socket Layer (HTTPS) connection realizing end-to-end encryption. Transport Layer Security 1.3 is used (1.2 is also supported), with no preference in cipher suite negotiation and an RSA 4096-bit (SHA256withRSA) certificate. The web server software that realizes the secure channel is Nginx [36], an open-source web server. The authentication and encryption algorithms are SHA-256 and AES (256-bit), respectively.

The experiments were planned as measurement sessions each considering one network setup (LAN, VPN, WAN) and one usage scenario (diagnostic data transfer, researcher or radiologist’s interaction). The tests were fully automated.

#### Measurements and Procedures

Every test and measurement procedure were implemented with automated tools to grant repeatability and exclude any human factor in the process. The whole testing procedure was also network-aware, as the system detects possible connection failures (repeating attempts up to a time-out) and route changes between test repetitions (ensuring that the whole sequence of repeated tests was conducted under an identical routing setup).

The test procedure for the diagnostic data transfer case comprised monitoring the resource consumption of all application modules while collecting the statistics generated by the “Docker stats” command for several days. These logs were analyzed by extracting numeric data to plot a time-series graph for CPU and memory usage.

**Figure 4:**
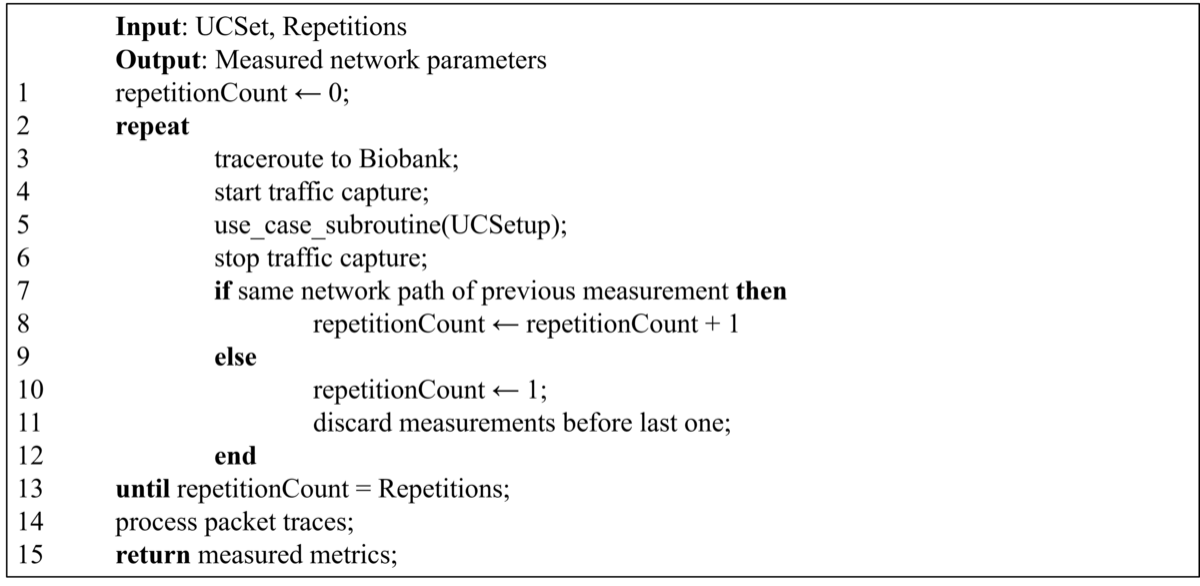
Algorithm 1: General web-based measurement procedure pseudocode. The use_case_subroutine() is implemented according to the specific use case.

The testing procedures for the researcher and radiologist access use cases are described as pseudocode in Algorithm 1 (see Figure 4). The difference between these two cases is accounted for in the use_case_subroutine() procedure (line 5). In both cases, this subroutine includes the log-in procedure (for each test repetition). This expedient better reproduces the human-operated nature of the task. It can be noted how each repetition begins with a trace route, and the resulting path is compared (line 7) with that collected in the previous repetition; if a change is detected, the number of repetitions already performed is reset to 1. In this way, the desired number of repetitions is guaranteed to occur in the same network path configuration, preventing routing changes during the repetitions from impacting the measured network performance. This is especially significant for the VPN and WAN setups, where traffic between the client and the biobank server traverses the public internet on which there is no control possibility. Network packet traces (lines 4 and 6) are captured during the user activity simulation for each repetition. These simulations must reproduce multiple actions of a human user, performed on the web interface (by clicking on the screen area and filling text fields). Therefore, the sequence of actions for these use cases have been captured from actual human interactions with the BCU-IB. To this aim, the human operators used a Chrome web browser instrumented with Selenium IDE and Chrome and Firefox plugins allowing recording and playback of user interactions with the browser. The recording of the user interaction was then converted in Python functional/acceptance tests that make use of Selenium WebDriver to reproduce human actions and grant repeatability of test cases.

**Figure 5:**
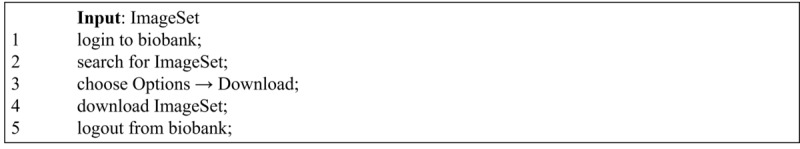
Algorithm 2: use_case_subroutine() pseudocode for the researcher use case.

**Figure 6:**
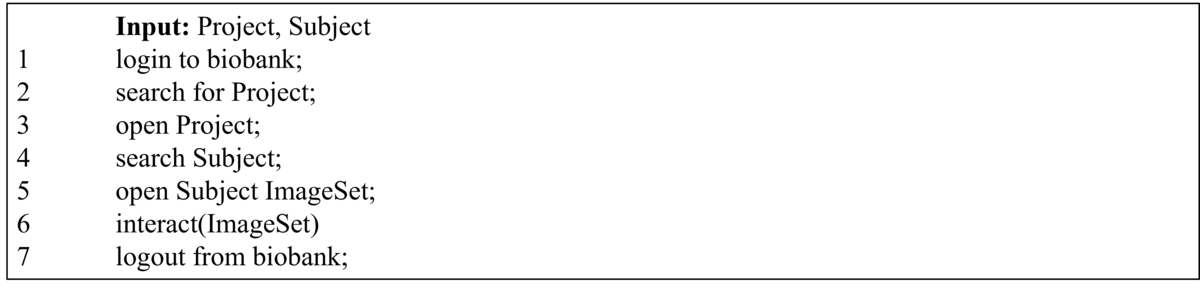
Algorithm 3: use_case_subroutine() pseudocode for the radiologist use case. Interact (ImageSet) includes a sequence of scrolling through slices and change of scan, with varying pace.

During the test execution, the Python script controlled a web browser and executed the case-specific use_case_subroutine(): the two series of user actions are described as pseudocode in Algorithm 2 for the researcher use case (Figure 5) and in Algorithm 3 for the radiologist use case (Figure 6).

All network traffic measurements were based on raw-packet traces captured on the client machine: the utility tcpdump was used to capture and filter traffic based on the biobank server internet protocol (all and only network traffic related to the client-server biobank communication is captured), while the tshark utility was used to extract network-related metrics.

The BCU-IB is a multitier application whose server component is implemented through multiple modules, each executed in its own lightweight virtualized environment (container). To evaluate the resource consumption (ie, resource footprint) associated with the interaction with the BCU-IB, the statistics of each involved container were collected. More specifically, CPU and memory usage monitoring was performed by collecting the full *docker stats* output at a 1-Hertz frequency. Moreover, application logs from each container were also collected.

## Results

### Diagnostic Data Transfer

**Figure 7:**
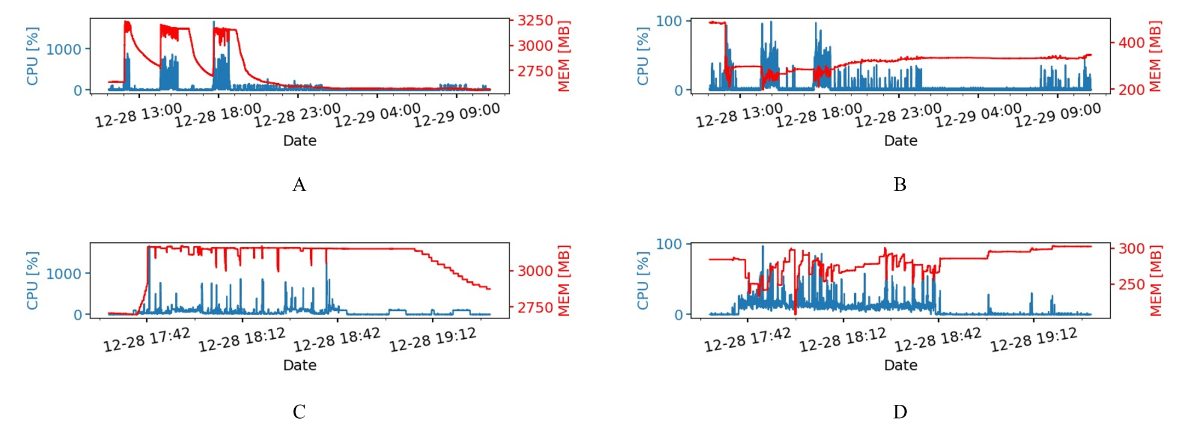
Time evolution of central processing unit (CPU; blue) and memory (MEM; red) usage during diagnostic data transfer for the Web (A) and Database (B) containers. (C and D) Relative maximized view of A and B, respectively.

Resource usage statistics were continuously collected for all the modules of the application (running as separate Docker containers). We found that the Nginx container (responsible for the SSL encryption in the WAN setup) showed negligible resource consumption and very little variability. Therefore, only results for the Web and Database containers are shown. Figure 7A and Figure 7B show the time evolution of resource consumption for one whole day (December 28, 2020) for the Web and Database containers. For both cases, a clear pattern can be seen, highlighting three activity spikes (corresponding to three upload events of several exams in the application log). Zoomed-in versions of the corresponding time series are shown in Figure 7C and Figure 7D, respectively, focusing on the third of such events (around 6 PM). These data demonstrated that the Web is the most memory-hungry container, averaging over 2.7 GB of RAM, with peaks of over 3 GB during the upload events. By contrast, the Database container only used an average of 300 MB, with no significant increases during the uploads (while CPU usage showed activations in correspondence with such events, rising from close to zero to ≈20% utilization). In addition, from the computing resources point of view, the results indicated that the Web container is the most demanding, with two sudden (but very short-lived) peaks at 1500% usage (corresponding to using 15 cores) and several peaks at slightly over 500%.

### Researcher and Radiologist Access

**Figure 8:**
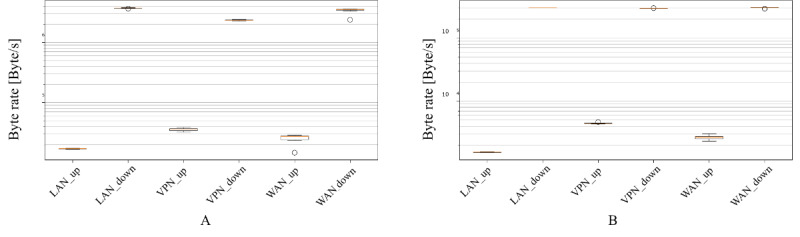
Quartile distribution of average throughput (over the whole duration of activity) for the researcher (A) and radiologist (B) use cases. LAN: local area network; VPN: virtual private network; WAN: wide area network.

Regarding the use cases reproducing the highly interactive access to the BCU-IB through the web interface, we analyzed both network performance and server-side resources consumption. Figure 8A and Figure 8B show the distribution of throughput for each of the network setups considered (LAN, VPN, WAN) in relation to the researcher and radiologist use case, respectively. It can be noted that for every case, the downstream direction of network traffic is almost two orders of magnitude higher than that of the upstream direction. This is coherent with the nature of the operations, mostly requiring the transfer of sizeable data (application dashboard, diagnostic images) in the server-client direction, as opposed to mostly commands in the client-server direction.

**Figure 9:**
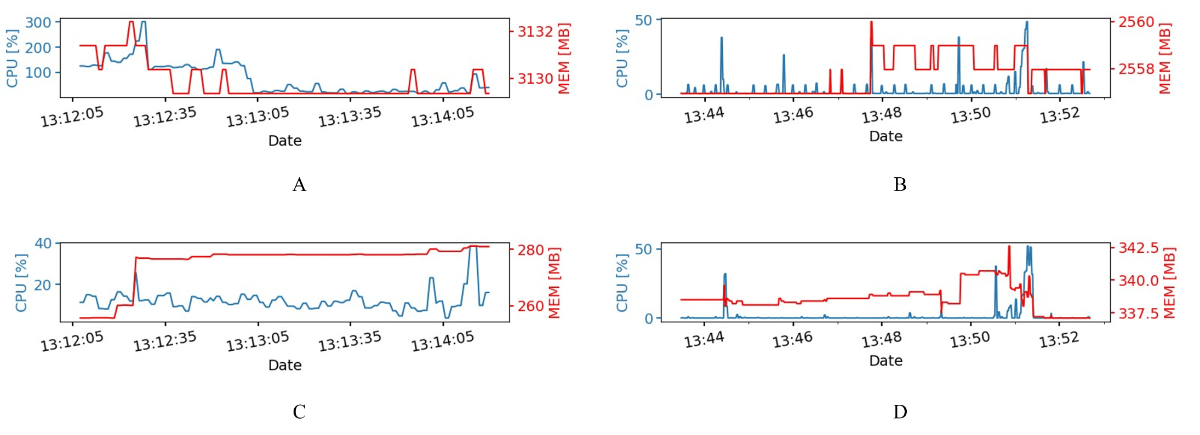
Time evolution of central processing unit (CPU; blue) and memory (MEM; red) usage for the Web (A) and Database (B) containers during the researcher use case reproduction and for the (C) Web (C) and Database (D) containers during the radiologist use case reproduction.

Regarding server resource consumption, user activities on the web client were logged and matched with server-side application and resource-monitoring logs based on the time stamp. Figure 9 shows the server-side resource consumption for both cases over time. As shown in Figure 9A, the most demanding operation took place in the first minute and corresponds to the image download phase, whereas Figure 9B shows negligible resource variation for the Database container. In Figure 9C and Figure 9D, it can be noted that download of the exam images is distributed across the entire test duration.

Tables 1 and 2 report resource consumption in terms of CPU usage for the main containers composing the application in correspondence to user (researcher/radiologist) operations causing notable variations. For each operation, the resource consumption is reported as the average value and total duration, along with small-duration peak consumption. The most resource-demanding operation was found to be DOWNLOAD & LOGOUT for the researcher case and the OPEN VIEWER operation (causing an automatic download of exam images) for the radiologist case. The Web container was involved for both users. In the latter case, we found not only a 15-second raised level but also a high peak for the Web and Database containers during 2 and 4 seconds, respectively. Moreover, the CHANGE SCAN operation caused peak consumption for such containers. In general, these results demonstrate that the current server setup is well-equipped to sustain more than a 10-fold increase of users concurrently performing the same kind of operation, and many more contemporary users can be served if their resource-demanding operations are not synchronized down to the same few-seconds interval.

**Table 1:** Server load and total duration during researcher interactions.

Operation	Resource consumption, %				Peak consumption, %				Total duration (seconds)
	Web	Database	Nginx	Web^a^		Database	Nginx		
Page Load	6	1	0	50		15	—^b^	2	
Login	6	2	0	25		—	—	2	
Filter & Search	6-10	2	0	15-25		30	—	2	
Download & Logout	100	0	5	—		24^c^	—	30-50	

^a^Sporadic case.

^b^Not analyzed owing to negligible consumption.

^c^The recorded peak lasted 2 seconds.

**Table 2:** Server load and total duration during radiologist interactions.

Operation	Resource consumption, %			Peak consumption, %				Total duration (seconds)
	Web	Database	Nginx	Web	Database	Nginx		
Page Load	6	1	0	50	15	—^a^	2	
Login	6	2	0	25	—	—	2	
Filter & Search	6-10	2	0	15-25	30	—	2	
Open Viewer^b^	20-50	20-50	0	114^c^	80^d^	—	15	
Change Scan	6	0	0	70-100	80-100	—	1	
Play/Stop^e^	6	—	—	20-34^c^	—	—	2-6	

^a^Not analyzed owing to negligible consumption.

^b^Exam images are downloaded at viewer opening.

^c^The recorded peak lasted 2 seconds.

^d^The recorded peak lasted 4 seconds.

^e^Mouse fast scroll.

## Discussion

In this work, we analyzed the network performance of the designed BCU-IB to both (1) assess the performance perceived by the operator in a realistic context and (2) evaluate the impact of the network technology, with the final goal of predicting the infrastructural requirements for providing the service. Multiple network scenarios have been considered, including the reference setup (LAN) and operational setups (VPN, WAN). The resource usage of the system was monitored and analyzed in relation to both diagnostic data transfer and interactive use by two kinds of biobank users. We focused on common operations performed by a researcher and a radiologist, highlighting the specific interactions that reflect actual undergoing analyses using the biobank in COVID-19 research. For the diagnostic data transfer use case, the results showed that the system is suitable to serve the planned number of users while maintaining a good reserve of resources. Indeed, on average, the application modules do not require more than 2 cores and 4 GB of memory during 1-hour-long data upload (Figure 9C-D), allowing at least a 10-fold increase of concurrent users. For the researcher and radiologist use cases, the results demonstrated that the most CPU-intensive interactive user operations (ie, OPEN VIEWER, CHANGE SCAN, DOWNLOAD & LOGOUT) allow for at least a 10-fold increase of concurrent users.

In our previous work [27], experiments were carried out to evaluate the network performance of the operational service considering three different types of setups (reference configuration with a single host, remote access via a VPN, and a LAN configuration). Two sets of images with different sizes (a set of medium-sized images of approximately 500 MB and a set of large-sized images of approximately 1 GB) were used to understand the impact of the internet path of all three network setups. This assessment was carried out considering the duration of the transfers in uploads and downloads; the amount of useful data transferred per unit of time, measured in MB/second; the average of this quantity per unit of time, measured in KB/second; the measure of the throughputs over time, measured in packets/second; and average of the number of throughputs, measured in bytes. Under the hypothesis that each single user experiences the network performance as in the measurement campaign, the results demonstrated that the system could accommodate more than 20 concurrent users for the upload usage case: for the planned intake of concurrent users (10 in the current stage of the service deployment), this amounted to an overprovisioning factor of 2. Regarding the download scenario, the ratio between server-side upstream bandwidth and client downstream byte rate was greater than 90, leading to an overprovisioning factor of 9. The current analysis confirmed these previous results. Hence, the operating system is validated for suitability for the planned number of concurrent users, also allowing for servicing exceptional cases, and offers ample time for planning and upscaling if the number of users admitted to the service is increased.

This benchmark is very useful for assessing the performance of the network under the hypothesis that the platform is used at full capacity as an imaging biobank by different users both in downloading and in uploading images. Nevertheless, this study has some limitations. Most notably, we did not investigate the scenario of concurrent usage by different users, and the overprovisioning of the analyzed system prevents the assessment of the minimum resources required for satisfying a basic level of service.

Further analyses will investigate performance scalability (for a sudden surge in concurrent users’ requests) both on the server and on the middleboxes traversed by the service traffic. An analysis of quality of experience, based on scores provided by practitioners in medical and clinical research, will also be performed. Moreover, ad hoc limitation of system resources will be applied to characterize the minimum requirements for providing a satisfying level of service.

## Abbreviations

BBMRI-ERIC: Biomolecular Resources Research Infrastructure-Education Information Resources Center
BCU: Bio Check Up Srl
BCU-IB: Bio Check Up Imaging Biobank
CPU: central processing unit
CT: computed tomography
DICOM: Digital Imaging and Communications in Medicine
HTTPS: Hypertext Transfer Protocol over Secure Socket Layer
ICT: information and communication technology
ISP: internet service provider
LAN: local area network
SAN: storage area network
SSL: secure socket layer
VPN: virtual private network
WAN: wide area network
XNAT: Extensible Neuroimaging Archive Toolkit

## Data Availability

The data generated in this study are available from the corresponding author on request.
